# Circulating Chromogranin B Is Associated With Left Ventricular Functional Recovery After Successful Recanalization of Chronic Total Occlusion

**DOI:** 10.3389/fcvm.2021.756594

**Published:** 2021-12-24

**Authors:** Ying Shen, Muladili Aihemaiti, Xin Yi Shu, Chen Die Yang, Jia Wei Chen, Yang Dai, Feng Hua Ding, Zhen Kun Yang, Jian Hu, Rui Yan Zhang, Lin Lu, Xiao Qun Wang, Wei Feng Shen

**Affiliations:** ^1^Department of Cardiovascular Medicine, Rui Jin Hospital, Shanghai Jiao-Tong University School of Medicine, Shanghai, China; ^2^Institute of Cardiovascular Diseases, Shanghai Jiao-Tong University School of Medicine, Shanghai, China

**Keywords:** chromogranin B, coronary collateralization, chronic total occlusion, left ventricular function, reverse remodeling

## Abstract

**Background:** Chromogranin B (CgB) is increased in heart failure and proportionate to disease severity. We investigated whether circulating CgB level is associated with left ventricular (LV) functional recovery potential after successful recanalization of chronic total occlusion (CTO).

**Methods:** Serum levels of CgB were assayed in 53 patients with stable angina with LV functional recovery [an absolute increase in LV ejection fraction (EF) of ≥5%] and 53 age- and sex-matched non-recovery controls after successful recanalization of CTO during 12-month follow-up.

**Results:** We found that CgB level was significantly lower in the recovery group than in the non-recovery group (593 [IQR 454–934] vs. 1,108 [IQR 696–2020] pg/ml, *P* < 0.001), and that it was inversely correlated with changes in LVEF (Spearman's r = −0.31, *P* = 0.001). Receiver operating characteristic (ROC) analysis showed that the area under the curve of CgB for predicting LVEF improvement was 0.76 (95% CI 0.664–0.856), and that the optimal cutoff value was 972.5 pg/ml. In multivariate analyses, after adjusting for confounding factors, high CgB level remained an independent determinant of impaired LV functional recovery after CTO recanalization. LV functional improvement appeared to be more responsive to CgB in patients with poor than with good coronary collaterals.

**Conclusions:** Elevated circulating CgB level confers an increased risk of impaired LV functional recovery after successful recanalization of CTO in patients with stable coronary artery disease.

## Introduction

Chronic total occlusion (CTO) occurs in 18–31% of patients with significant coronary artery disease undergoing routine coronary angiography (1–3). Both randomized trials and observational studies have demonstrated that successful revascularization of CTO lesions accomplished by percutaneous coronary intervention (PCI) or coronary artery bypass grafting is associated with a number of clinical benefits, such as anginal symptom relief, improved quality of life and left ventricular (LV) function, and decreased mortality when compared to CTO patients whose recanalization failed or those who received optimal medical treatment only (4–7). Therefore, revascularization is recommended as an initial therapeutic modality in patients with CTO by current guidelines (8). The presence of severe obstructive or occluded coronary lesions has been considered a prerequisite for spontaneous collateral recruitment (9). Coronary collaterals provide salvage of ischemic myocardium and preserve cardiac function in patients with coronary artery disease and CTO (10), and patients with robust collaterals are more likely to have successful revascularization of CTO and experience lower risk of mortality and adverse cardiovascular events compared with those with poor or undetectable collaterals (11). Nevertheless, knowledge of factors stratifying the recovery potential of LV function or collateral conditions in patients with CTO is still lacking.

Chromogranin B (CgB), also known as secretogranin I, is a tyrosine-sulfated protein widely expressed in secretory granules of endocrine, neuroendocrine cells, and cardiomyocytes (12–14). In cardiomyocytes, CgB functions as a novel regulator of brain natriuretic peptide (BNP) through InsP3/Ca^2+^-dependent signaling (15). In animals and patients with heart failure, circulating CgB level was found to be significantly increased, and was in proportion to disease severity (16). Remarkably, CgB, better than NT-proBNP, discriminates clinical functional status of patients with chronic heart failure. However, reports on circulating CgB levels in association with other cardiovascular disease are scarce, especially the relationship between serum CgB and LV function in patients with stable coronary artery disease and CTO remains unclear. In this study, we sought to investigate the association between serum CgB level and subsequent LV functional recovery after successful recanalization of CTO lesions in relation to the status of coronary collateralization.

## Methods

### Study Population

**Figure 1:**
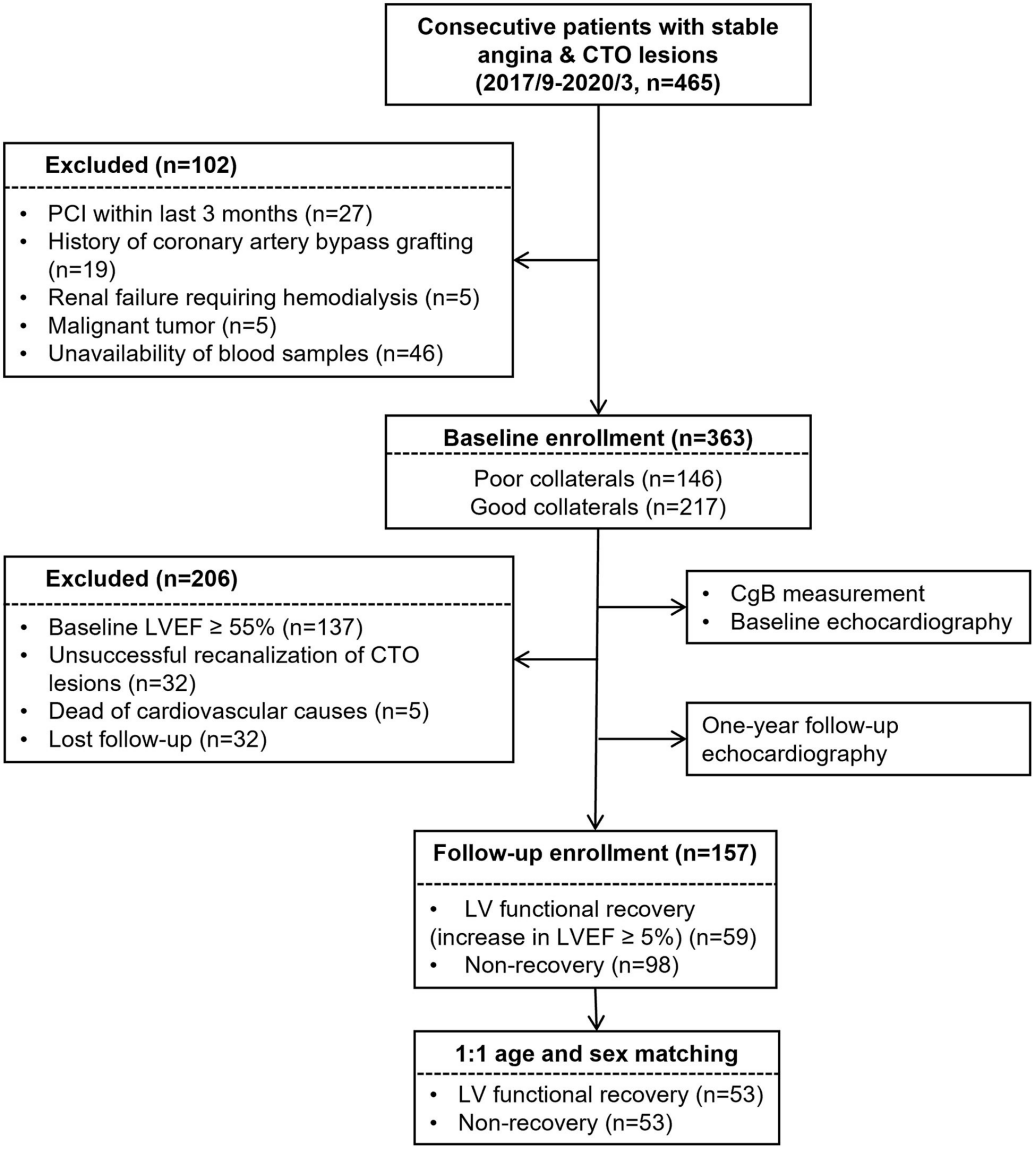
Flowchart of patient enrollment.

A total of 465 consecutive patients with stable angina and CTO (>3 months) of at least one major epicardial coronary artery between September 2017 and May 2019 were screened from the database of Shanghai Rui Jin Hospital PCI Outcomes Program. The duration of coronary artery occlusion was estimated from the date of occurrence of myocardial infarction in the area supplied by the occluded vessel, from abrupt worsening of existing angina pectoris, or from information obtained from a previous angiogram. For study purpose, 56 patients were excluded because of recent PCI within last 3 months (*n* = 27), history of coronary artery bypass grafting (*n* = 19), renal failure requiring hemodialysis (*n* = 5), and malignant tumor (*n* = 5), as these conditions could influence collateral formation. Forty-six patients were further excluded because of unavailability of blood samples. Thus, 363 patients were enrolled and underwent baseline echocardiography. Due to the study purpose of LV functional recovery, we excluded 137 patients with baseline LV ejection fraction (LVEF) ≥55%. Additional 32 patients were excluded because of unsuccessful CTO recanalization. The cohort was followed up for around 12 months and underwent repeat echocardiographic examination. There were 5 patients who died from any cause, and 32 got lost to follow-up. Afterward, 157 patients were kept, and 53 pairs of age- and sex-matched patients with or without LV functional recovery were enrolled in the final analysis (Figure 1).

A diagnosis of type 2 diabetes mellitus was made according to the criteria of American Diabetes Association [symptoms of diabetes with casual plasma glucose concentration ≥200 mg/dl [11.1 mmol/L] or fasting plasma glucose ≥126 mg/dl [7 mmol/L], 2-h postprandial glucose ≥200 mg/dl [11.1 mmol/L] during oral glucose tolerance test, and currently or previously treated with insulin and/or oral hypoglycemic agents] (17). Hypertension was diagnosed according to the seventh report of the Joint National Committee on prevention, detection, evaluation, and treatment of high blood pressure (JNC 7) (18).

The protocol of the study was approved by the Institutional Review Board of Rui Jin Hospital, Shanghai Jiao Tong University School of Medicine, and clinical investigation was conducted according to the principle of the Declaration of Helsinki. Written informed consent was obtained from all the patients.

### Biochemical Assessments

Blood samples were obtained on the day of angiography from all the patients after overnight fasting. To avoid a diurnal variation in CgB concentration and dramatic fasting interval effects, all blood samples were obtained at 8:00 am. Serum levels of creatinine, lipid profiles, glucose, glycosylated hemoglobin A_1c_ (HbA_1c_), and N-terminal pro-B-type natriuretic peptide (NT-proBNP) were determined with standard laboratory techniques. Estimated glomerular filtration rate (eGFR) was computed using the Chronic Kidney Disease Epidemiology Collaboration equation. Serum CgB was assayed using an enzyme-linked immunosorbent assay (ELISA) kit (Cat# NBP2-75273; Novus Biologicals, Littleton, CO, United States).

### Angiography and Collateral Grading

Coronary angiography was performed through the femoral or radial access with 6 Fr diagnostic catheters. All the angiograms were analyzed independently by two blinded interventional cardiologists. The degree of coronary artery disease was assessed according to the lesion classification scheme of the American College of Cardiology/American Heart Association. The presence and degree of coronary collateralization from the contralateral vessel (often *via* connections of the epicardial surface or intraventricular septum) was visually estimated using the Rentrop grading system (19): 0 = no visible filling of any collateral channel; 1 = filling of side branches of the artery to be perfused by collateral vessels without visualization of epicardial segment; 2 = partial filling of the epicardial artery by collateral vessels; 3 = complete filling of the epicardial artery by collateral vessels. The patients were categorized into poor (grade 0 or 1) or good (grade 2 or 3) coronary collateralization group. This angiographic assessment of coronary collaterals is routinely applied in clinical practice. In case of disagreement, the difference in interpretation was resolved by a third reviewer.

### Echocardiographic Examination

Comprehensive transthoracic echocardiography was performed within 48 h before coronary angiography/intervention and at 12-month follow-up using a commercially available system (Vivid-I; GE Healthcare, Milwaukee, WI, United States) with a 1.9–3.8-mHz phased-array transducer by a single sonographer credentialed in cardiac ultrasound. Two-dimensional echocardiography and Doppler flow imaging were recorded from standard parasternal and apical transducer positions with a frame rate of 60–100 frames/s. All data were stored digitally, and an offline analysis was performed (EchoPac, version 7; GE Healthcare, Milwaukee, WI, United States) by two independent experienced cardiologists at the end of the study blinded to the study time point.

Left ventricular (LV) end diastolic and end systolic volumes, and LVEF were measured using the modified Simpson's biplane technique in a cardiac cycle not preceded or followed by a premature complex, according to the recommendations of the American Society of Echocardiography and the European Association of Cardiovascular Imaging for cardiac chamber quantification by echocardiography in adults (19). To facilitate the application of clinical normality cut points, LV end diastolic and end systolic volumes were indexed (i.e., EDVI and ESVI) by body surface area using the formula 0.061 × height + 0.0128 × weight−0.1529. LV mass was estimated from M-mode measurements with the formula LV mass = 0.8× 1.04 × [(*LVEDD* + *IVST* + *LVPWT*)^3^ − *LVEDD*^3^]+0.6, where LVEDD is LV end-diastolic diameter, IVST is interventricular septal thickness, and LVPWT is LV posterior wall thickness. LV mass was indexed by BSA. LV function recovery was defined as an absolute increase in LVEF of ≥5% 1 year after recanalization of CTO lesions.

### Statistical Analyses

Continuous variables are presented as median (interquartile range) or mean ± standard deviation (SD), and categorical data are summarized as frequencies (percentages). The normal distribution of continuous variables was evaluated by Shapiro-Wilk test. For normally distributed variables, differences in serum CgB were compared by Student's *t*-test. For non-normally distributed continuous variables, differences were analyzed by Mann-Whitney *U*-test. Differences in categorical variables were analyzed by χ^2^-test. The correlations between CgB, NT-proBNP, LVEF and their changes were analyzed by Spearman's correlation test. The diagnostic value of CgB was calculated by constructing a receiver-operating characteristic (ROC) curve, and the optimal cutoff threshold was determined by Youden's index. Three models were constructed in the multivariate analysis. In model I, all conventional risk factors and significant predictors were enrolled followed by backward stepwise elimination. Afterward, CgB level (model II), collateral conditions and their interaction term with CgB were further included (model III). Restricted cubic spline analysis was performed to evaluate the relationship between CgB levels and LV functional recovery under different collateral conditions after multivariate adjustment. Net reclassification improvements (NRIs) and integrated discrimination improvements (IDIs) were analyzed to assess the improvement in clinical utility of the prediction model by considering CgB. All the statistical analyses were performed using the R statistical package version 4.0.3 (R Project for Statistical Computing, Vienna, Austria). A 2-tailed *p* < 0.05 was considered statistically significant.

## Results

### Baseline Characteristics of the Study Population

Patients with or without LV functional recovery after CTO recanalization did not differ with respect to history of hypertension, diabetes, previous myocardial infarction, smoking habits, blood pressure, renal function, and medical treatments. The distribution of CTO lesions and collateral conditions were similar between the 2 groups. Interestingly, patients with LV functional recovery had lower fasting glucose but higher total and LDL cholesterol levels than non-recovery subjects. Serum levels of NT-proBNP were also comparable between the 2 groups (Table 1).

**Table 1:** Baseline demographic and clinical characteristics.

	**Non-recovery**	**Recovery**	***P*** **-value**
***n***	**53**	**53**	
**Demographic characteristics and clinical assessments**			
Male sex	48 (90.6)	48 (90.6)	1.000
Age, years	59.92 ± 8.90	57.62 ± 11.95	0.263
Hypertension	25 (47.2)	23 (43.4)	0.845
Diabetes	28 (52.8)	30 (56.6)	0.845
Previous myocardial infarction	6 (11.3)	11 (20.8)	0.290
Smoking habits	27 (50.9)	35 (66.0)	0.168
BMI, kg/m^2^	24.87 ± 4.45	24.38 ± 2.65	0.493
Systolic BP, mmHg	132.92 ± 22.77	125.36 ± 17.18	0.056
Diastolic BP, mmHg	77.47 ± 17.00	73.34 ± 10.47	0.135
**Laboratory measurements**			
HbA1c, %	6.58 ± 1.31	6.38 ± 1.37	0.456
Fasting glucose, mmol/L	6.29 (5.01–7.82)	5.10 (4.97–5.84)	0.009
Fasting insulin, μU/L	8.02 (6.24–10.64)	6.83 (5.58–13.82)	0.663
HOMA-IR	2.15 (1.60–4.27)	1.71 (1.32–3.79)	0.096
Triglyceride, mmol/L	1.50 (1.20–3.56)	1.54 (1.09–1.85)	0.277
Total cholesterol, mmol/L	4.08 ± 1.14	4.65 ± 1.14	0.011
HDL cholesterol, mmol/L	1.02 ± 0.20	0.97 ± 0.20	0.190
LDL cholesterol, mmol/L	2.33 ± 0.85	3.04 ± 0.98	<0.001
Serum creatine μmol/L	115.13 ± 147.79	78.45 ± 15.47	0.075
Blood urea nitrogen, mmol/L	7.36 ± 5.39	6.21 ± 2.60	0.164
eGFR, mL/min/1.732 m^2^	95.18 ± 19.79	101.03 ± 15.07	0.090
hsCRP, mg/L	0.64 (0.48–1.35)	1.30 (0.75–14.00)	0.188
NT-proBNP, pg/mL	389.40 (247.00–701.50)	377.50 (208.80–667.10)	0.756
CgB, pg/mL	1107.56 (695.54–2020.43)	592.71 (454.37–934.33)	<0.001
**Lesion characteristics**			
Multivessel disease	35 (66.0)	41 (77.4)	0.281
CTO vessels			
LAD	20 (37.7)	15 (28.3)	0.567
LCX	18 (34.0)	22 (41.5)	
RCA	24 (45.3)	25 (47.2)	
Good collaterals (Rentrop grade 2–3)	25 (47.2)	15 (28.3)	0.071
**Medication use**			
Aspirin	47 (88.7)	51 (96.2)	0.270
P2Y_12_ inhibitor	50 (94.3)	53 (100.0)	0.241
Beta blocker	41 (77.4)	40 (75.5)	1.000
ACEI/ARB	25 (47.2)	37 (69.8)	0.030
Calcium channel blocker	7 (13.2)	7 (13.2)	1.000
Spironolactone	14 (26.4)	13 (24.5)	1.000

*ACEI, angiotensin-converting enzyme inhibitor; ARB, angiotensin receptor blocker; BMI, body mass index; BP, blood pressure; CgB, chromogranin B; CTO, chronic total occlusion; eGFR, estimated glomerular filtration rate; HbA_1c_, glycated hemoglobin A_1c_; HDL, high-density lipoprotein; HOMA-IR, homeostatic model assessment for insulin resistance; hsCRP, high-sensitivity C-reactive protein; LDL, low-density lipoprotein*.

### LV Function and Geometric Changes After CTO-PCI

Before PCI, patients with and without LV functional recovery had similar LV volumes (EDVI: *P* = 0.062; ESVI: *P* = 0.409) and LVEF (*P* = 0.647). During follow-up, mean increase in LVEF was 9.91 ± 3.52% in the recovery vs. 0.19 ± 2.99% in the non-recovery group. Pronounced LV reverse remodeling was observed in the recovery rather than non-recovery patients (ΔEDVI: −1.48 ± 5.87 vs. 2.53 ± 10.67 ml/m^2^, *P* = 0.018; ΔESVI: −5.47 ± 4.58 vs. 1.82 ± 7.64, *P* < 0.001) (Table 2, Supplementary Table 1).

**Table 2:** Changes in left ventricular volume and ejection fraction during follow-up.

**Parameters**		**Non-recovery** **(***n*** = 53)**	**Recovery** **(***n*** = 53)**	***P*** **-value**
EDVI, mL/m^2^	B	90.52 ± 27.70	81.31 ± 22.24	0.062
	F	93.05 ± 30.02	79.83 ± 21.36	0.010
	Δ	2.53 ± 10.67	−1.48 ± 5.87	0.018
ESVI, mL/m^2^	B	46.45 ± 20.00	43.17 ± 20.77	0.409
	F	48.28 ± 21.92	37.71 ± 19.20	0.010
	Δ	1.82 ± 7.64	−5.47 ± 4.58	<0.001
Ejection fraction, %	B	47.15 ± 6.84	46.45 ± 8.71	0.647
	F	47.34 ± 7.53	56.36 ± 8.75	<0.001
	Δ	0.19 ± 2.99	9.91 ± 3.52	<0.001

*B, baseline; Δ, changes in corresponding parameters; F, follow-up; EDVI, left ventricular end-diastolic volume index; ESVI, left ventricular end-systolic volume index*.

### Relationship Between CgB and LV Functional Recovery

**Figure 2:**
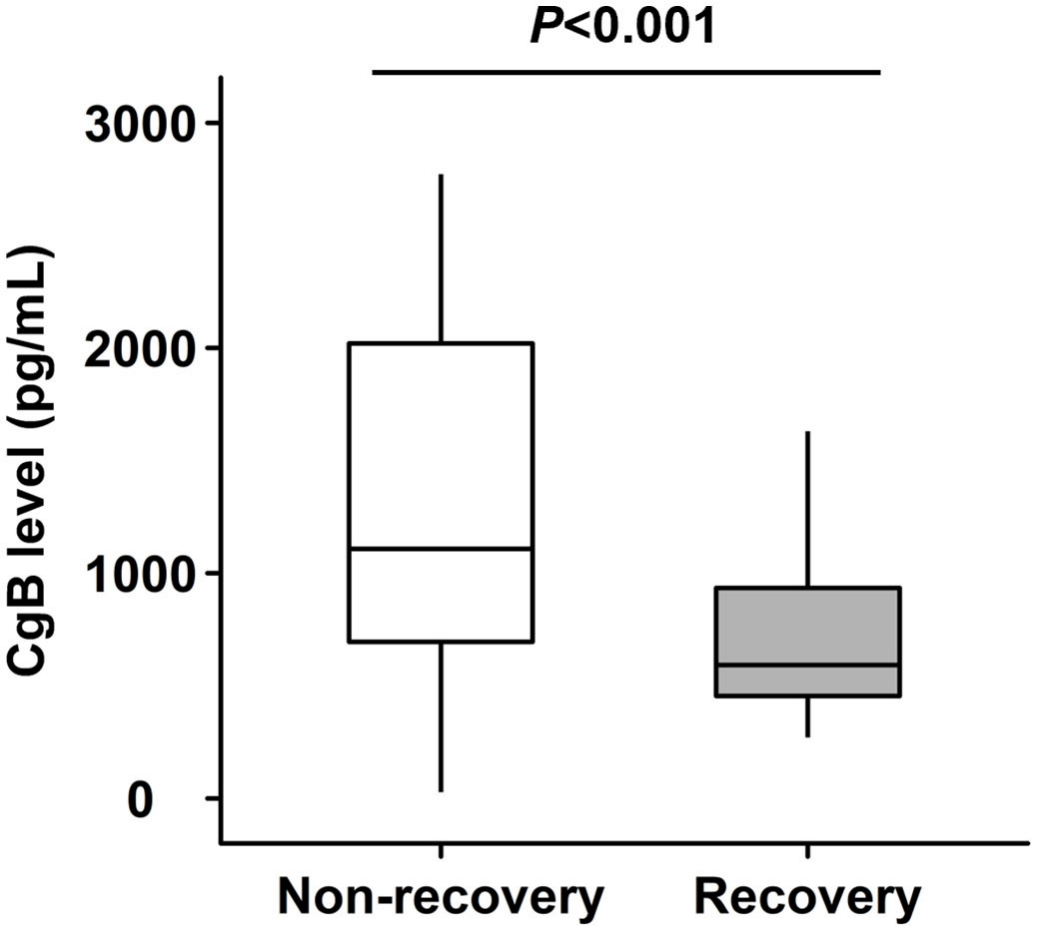
Levels of chromogranin B (CgB) in patients with or without left ventricular (LV) functional recovery after recanalization of chronic total occlusion (CTO) lesions.

**Figure 3:**
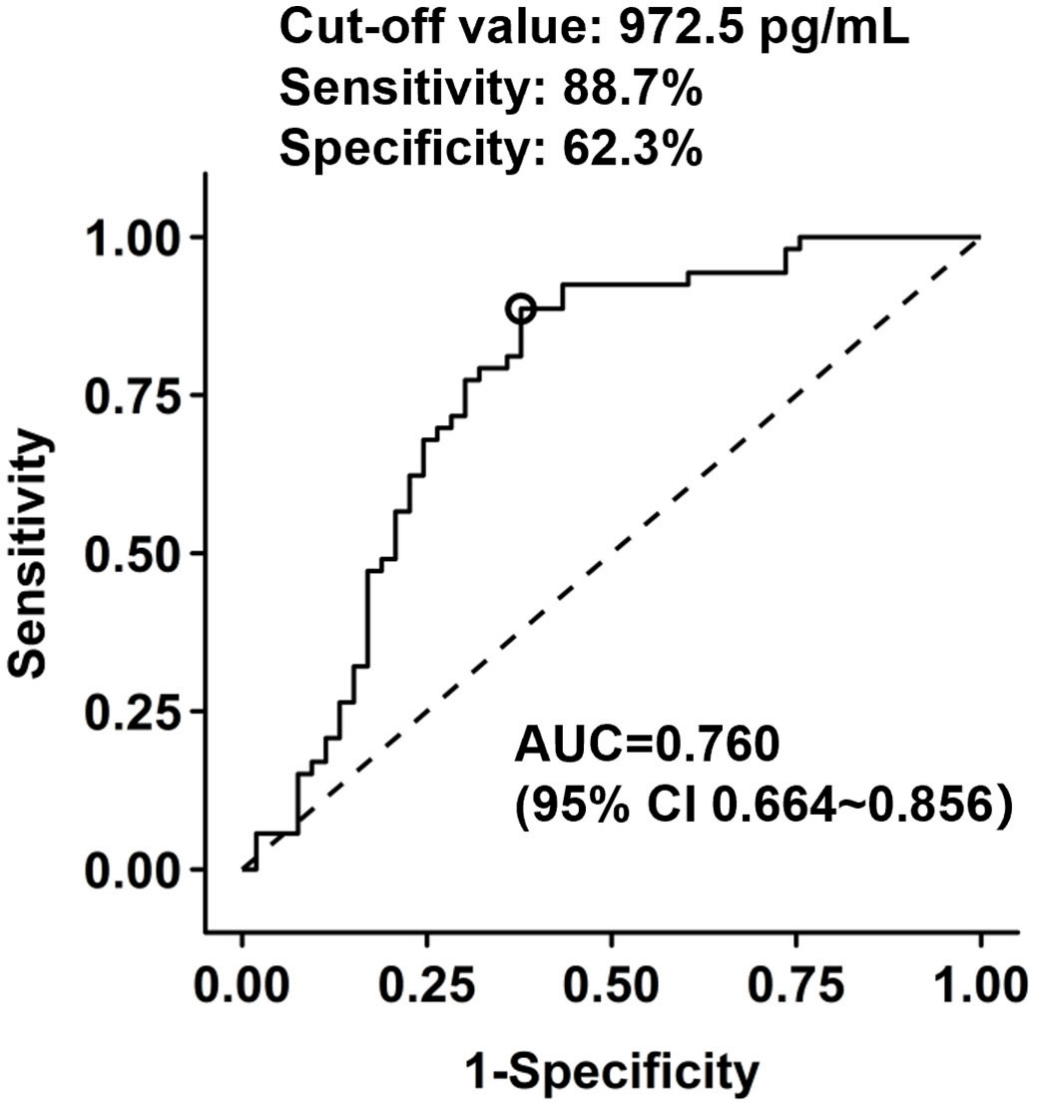
Receiver operating characteristic (ROC) analysis of chromogranin B levels in prediction of LV functional recovery after CTO recanalization.

Patients with LV functional recovery had significantly lower CgB level than non-recovery patients (593 [IQR 454–934] vs. 1,108 [IQR 696–2,020] pg/ml, *P* < 0.001, Figure 2) at baseline. CgB level was inversely correlated to changes in LVEF (Spearman's r = −0.31, *P* = 0.001) during the 1-year follow-up but not to baseline LVEF (Spearman's r = −0.08, *P* = 0.416) or NT-proBNP levels (Spearman's r = −0.04, *P* = 0.687). ROC curve analysis showed that the area under the curve (AUC) of CgB for predicting LV functional recovery after CTO-PCI was 0.76 (95% CI 0.664–0.856), and that the optimal cutoff threshold was 972.5 pg/ml, which corresponded to a diagnostic sensitivity of 88.7% and specificity of 62.3% (Figure 3).

### Multivariate Analysis

**Figure 4:**
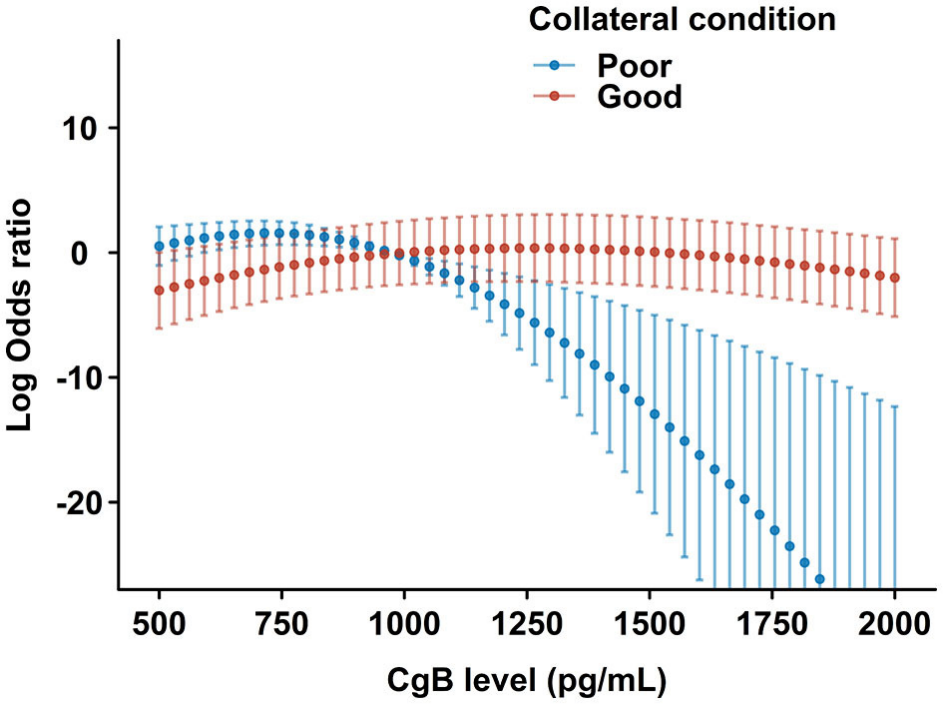
Restricted cubic spline plots for the likelihood of LV functional recovery by CgB levels after multivariate adjustment. The central dots and error bars represent the adjusted odds ratios and 95% confidence intervals in patients with poor (blue) or good (red) collateral conditions after CTO- percutaneous coronary intervention (PCI).

Higher CgB levels were associated with impaired LV functional recovery after CTO-PCI during the follow-up (OR: 0.853 [95% CI 0.785–0.913]). Multivariate analysis (Table 3) revealed that higher eGFR (OR: 1.325 [95% CI 1.022–1.777]) and cholesterol levels (OR: 1.541 [95% CI 1.063–2.283]), lower BMI (OR: 0.876 [95% CI 0.773–0.986]) and EDV index (OR: 0.824 [95% CI 0.685–0.979]), and use of RAAS inhibitors were significantly associated with LV functional recovery after CTO-PCI (model I). After inclusion of CgB level in the model (model II), it remained inversely associated with LV functional recovery (OR: 0.832 [95% CI 0.760–0.895]). There was a significant interaction term (*P* = 0.037) between CgB level and collateral conditions (model III). Patients with poor collaterals appeared to be more responsive to CgB levels with regard to LV functional recovery than those with good collaterals (Figure 4).

**Table 3:** Multivariate analysis.

**Variates**	**Model I**		**Model II**		**Model III**	
	**OR (95% CI)**	***P*** **-value**	**OR (95% CI)**	***P*** **-value**	**OR (95% CI)**	***P*** **-value**
BMI	0.876 (0.773–0.986)	0.031	0.895 (0.752–1.039)	0.174	0.774 (0.626–0.934)	0.011
eGFR, per 10 mL/min/1.732 m^2^	1.325 (1.022–1.777)	0.044	1.439 (1.025–2.186)	0.055	1.390 (1.005–2.060)	0.066
Cholesterol, per mmol/L	1.541 (1.063–2.283)	0.026	1.787 (1.160–2.885)	0.011	2.320 (1.398–4.171)	0.002
ACEI/ARB	3.763 (1.524–9.908)	0.005	10.149 (3.238–39.393)	<0.001	7.934 (2.334–32.095)	0.002
EDVI, per 10 ml/m^2^	0.824 (0.685–0.979)	0.033	0.786 (0.627–0.961)	0.025	0.777 (0.613–0.956)	0.024
CgB, per 100 pg/mL			0.832 (0.760–0.895)	<0.001	0.699 (0.544–0.831)	0.001
Collateral conditions					0.109 (0.007–1.192)	0.090
CgB × Collateral conditions					1.338 (1.056–1.821)	0.037

*ACEI, angiotensin-converting enzyme inhibitor; ARB, angiotensin receptor blocker; BMI, body mass index; CgB, chromogranin B; EDVI, left ventricular end-diastolic volume index; eGFR, estimated glomerular filtration rate*.

The inclusion of CgB led to better prediction accuracy. In the non-recovery patients, 29.1% was correctly reclassified to lower probability categories, and 10.9% was reclassified to higher categories. In patients with LV functional recovery, 21.4% was correctly reclassified to higher probability categories, and none was reclassified to lower probability categories. The categorical NRI was 39.61% (95% CI: 20.33–58.89%, *P* < 0.001), and IDI was 23.38% (95% CI: 15.44–31.31%, *P* < 0.001).

## Discussion

The results of this study show that circulating CgB level stratified LV functional recovery potential after successful recanalization of CTO lesions. Patients with CgB ≥ 972.5 pg/ml were unlikely to benefit from CTO-PCI with regard to LV functional recovery. The association between CgB levels and LVEF improvement was affected by collateral conditions.

Currently, with advances in dedicated device, technical strategy, and interventional skills, the overall success rate of CTO-PCI has been dramatically increased (20, 21). Successful CTO-PCI is generally believed to bring favorable effects on clinical outcomes and quality of life. A number of studies have shown that CTO recanalization and patency of the occluded arteries confer improvement in LV global and regional functions (22–25), especially in patients without previous myocardial infarction (24). However, the RESVASC trial showed no benefit from CTO-PCI with regard to multiple indices of LV function despite significant reduction in major adverse cardiovascular events at 12 months (26). In this study, a significant portion of patients had increased LVEF and LV reverse remodeling at 12-month follow-up after CTO-PCI, and this recovery process seems to be conditional on multiple systemic and local factors such as circulating CgB levels and coronary collateral conditions.

Delicate studies utilizing cardiac magnetic resonance (7), positron emission tomography (27), and intracoronary flow and pressure measurements (22) suggest that myocardial viability, microvascular integrity, and collateral conditions are determinants of LV functional recovery potential after CTO-PCI (28). Our study, for the first time, reported that a novel serum marker, CgB, is an independent predictor of LV functional recovery after CTO-PCI. The association between CgB and LVEF improvement was affected by collateral conditions. LV functional recovery appeared to be more responsive to CgB in patients with poor than with good collaterals. Although the underlying mechanism is still unclear, a possible explanation is that low CgB may reflect a good compensatory function of the global left ventricle even in the presence of a portion of ischemic myocardium due to poor collaterals from the donor artery, and, thus, better recovery potential after CTO recanalization.

Previous studies have demonstrated that CgB was markedly elevated in patients and animal models with heart failure, and was in proportion to disease severity (15, 16). Here, we further showed that CgB was significantly and inversely related to changes in LVEF after successful CTO-PCI. Interestingly, there was no association between CgB and LVEF and NT-proBNP levels at baseline. It might be due to the fact that a great portion of the enrolled patients (82.1%) had moderately but not severely reduced LVEF, and the association between LV function and CgB could be further complicated by the potential influence of the size of myocardial territories subtended by the occluded artery and collateral conditions on CgB levels. These data provide evidence that CgB is not only associated with LV dysfunction but also with LV functional recovery potential.

Noteworthy is that the inverse relationship 0 between CgB and LV functional recovery is presumably complicated by the net synergistic and antagonistic effects of CgB on other types of chromogranins, degraded peptides, and downstream products. CgB is often co-localized with chromogranin A, a recently discovered cardiac biomarker. Ceconi et al. found that chromogranin A has high sequence and function similarities as CgB, and that increased chromogranin independently predicted mortality in patients with chronic heart failure (29). Likewise, we have previously demonstrated that decreased vasostatin-2, a peptide derived from chromogranin A possessing pronounced anti-inflammatory and anti-atherogenic actives, was associated with chronic heart failure and incidence of major adverse cardiac events during a 3-year follow-up (30). These observations suggest that further studies by cytokine quantification and *in vitro* experiments are required to depict the precise role of CgB and related peptides in the regulation of LV function and reserve capacity for patients with stable coronary artery disease and CTO, especially after successful revascularization.

Our findings should be interpreted in the context of the following limitations: first, this was a single center, retrospective study that had a small sample size, which might diminish the power of the drawn statistical inference. A further study with a large scale prospective, multicenter design is, thus, required to confirm our results. Second, the Rentrop scale of angiographic collateral grading is semi-quantitative, whereas the assessment of collateral flow using a qualitative method should be more precise (10). Third, all the study populations were Chinese; thus, caution should be paid when extrapolating the study findings to other ethnic groups. Fourth, as illustrated above, both chromogranins and their corresponding degraded peptides have regulatory activities in a variety of cardiovascular diseases; therefore, concurrent measurement of CgB and its degraded peptide could provide more insights into the regulatory mechanisms.

## Conclusions

This study suggests that elevated circulating CgB level is a surrogate biomarker of impaired LV functional recovery potential after successful recanalization of CTO lesions in patients with stable coronary artery disease, especially for those with poor collaterals. Further studies are warranted to investigate the impact of circulating CgB on decision-making for revascularization strategy and evaluation of clinical outcomes in these patients.

## Data Availability Statement

The raw data supporting the conclusions of this article will be made available by the authors, without undue reservation.

## Ethics Statement

The studies involving human participants were reviewed and approved by Ruijin Hospital. The patients/participants provided their written informed consent to participate in this study.

## Author Contributions

YS, XW, LL, and WS: study conception and design. YS, MA, XS, CY, JC, MA, FD, ZY, JH, and XW: acquisition and analysis of data. CY, MA, and XS: blood sample management, preparation, ELISA, and analysis. YD and RZ: drafting of a significant portion of the manuscript. XW, LL, and WS: revision of the manuscript for important intellectual content. All authors contributed to the article and approved the submitted version.

## Funding

This study was supported by the National Natural Science Foundation of China (Grant Nos: 82170423, 81870179, 8200369, 81770430, 81770437, and 81770447), Shanghai Municipal Commission of Health and Family Planning (Grant Nos: 2018YQ17 and 20194Y0042), Shanghai Science and Technology Commission Natural Fund Project (17ZR1417200), Shanghai High School Fellowship Program for Research & Translation (Grant No: RC0030103), Ruijin Youth Training Program (Grant No: 2019QNPY01033), and Shanghai Municipal Education Commission-Gaofeng Clinical Medicine Grant Support (20181801).

## Conflict of Interest

The authors declare that the research was conducted in the absence of any commercial or financial relationships that could be construed as a potential conflict of interest.

## Publisher's Note

All claims expressed in this article are solely those of the authors and do not necessarily represent those of their affiliated organizations, or those of the publisher, the editors and the reviewers. Any product that may be evaluated in this article, or claim that may be made by its manufacturer, is not guaranteed or endorsed by the publisher.
