# Supplementary material for: Circulating Chromogranin B Is Associated With Left Ventricular Functional Recovery After Successful Recanalization of Chronic Total Occlusion
Source: Front Cardiovasc Med. 2021 Dec 24;8:756594. doi: 10.3389/fcvm.2021.756594 (PMC8740892; doi:10.3389/fcvm.2021.756594)
Supplement: Supplementary file 1 [file Table_1.pdf]

ONLINE SUPPLEMENTS

Supplementary table 1. Changes in M-mode echo measurements during follow-up

		Non-recovery （n=53）	Recovery (n=53)	P-value
**LVEDD, mm**	B	56.94±7.45	53.92±6.64	0.030
	F	57.53±7.20	53.58±6.17	0.003
	Δ	0.58±2.79	-0.34±1.63	0.040
**LVESD, mm**	B	42.25±7.85	40.42±8.16	0.242
	F	42.85±7.59	38.17±8.03	0.003
	Δ	0.60±2.72	-2.25±2.29	<0.001
**LV mass index, g/m^2**	B	10.38±1.70	9.68±1.11	0.014
	F	10.30±1.45	9.57±1.38	0.009
	Δ	-0.08±1.07	-0.11±1.07	0.856
**IVST, mm**	B	9.04±0.83	8.64±0.83	0.016
	F	9.32±0.96	8.60±0.93	<0.001
	Δ	0.28±0.95	-0.04±0.65	0.045
**PWT, mm**	B	119.78±29.46	104.14±25.76	0.004
	F	124.13±32.57	102.04±25.07	<0.001
	Δ	4.35±19.61	-2.10±9.78	0.034

B, baseline; Δ, changes in corresponding parameters; F, follow-up; LVEDD, left ventricular end-diastolic diameter; LVESD, left ventricular end-systolic diameter; IVST, interventricular septal thickness; PWT, left ventricular posterior wall thickness.
